# Enhanced lung cancer detection: Integrating improved random walker segmentation with artificial neural network and random forest classifier

**DOI:** 10.1016/j.heliyon.2024.e29032

**Published:** 2024-04-04

**Authors:** Sneha S. Nair, V.N. Meena Devi, Saju Bhasi

**Affiliations:** a Department of Physics, Noorul Islam Centre for Higher Education, Kumarakovil, Kanyakumari District, Tamil Nadu, India; b Department of Radiation Physics, Regional Cancer Centre, Thiruvananthapuram, Kerala, India

**Keywords:** Accuracy, Classifier, Computed tomography, Diagnosis, LIDC dataset, Lung cancer, Random forest

## Abstract

**Background:**

Medical image segmentation is a vital yet difficult job because of the multimodality of the acquired images. It is difficult to locate the polluted area before it spreads.

**Methods:**

This research makes use of several machine learning tools, including an artificial neural network as well as a random forest classifier, to increase the system's reliability of pulmonary nodule classification. Anisotropic diffusion filtering is initially used to remove noise from a picture. After that, a modified random walk method is used to get the region of interest inside the lung parenchyma. Finally, the features corresponding to the consistency of the picture segments are extracted using texture-based feature extraction for pulmonary nodules. The final stage is to identify and classify the pulmonary nodules using a classifier algorithm.

**Results:**

The studies employ cross-validation to demonstrate the validity of the diagnosis framework. In this instance, the proposed method is tested using CT scan information provided by the Lung Image Database Consortium. A random forest classifier showed 99.6 percent accuracy rate for detecting lung cancer, compared to a artificial neural network's 94.8 percent accuracy rate.

**Conclusions:**

Due to this, current research is now primarily concerned with identifying lung nodules and classifying them as benign or malignant. The diagnostic potential of machine learning as well as image processing approaches are enormous for the categorization of lung cancer.

## Graphical abstract

**Figure:**
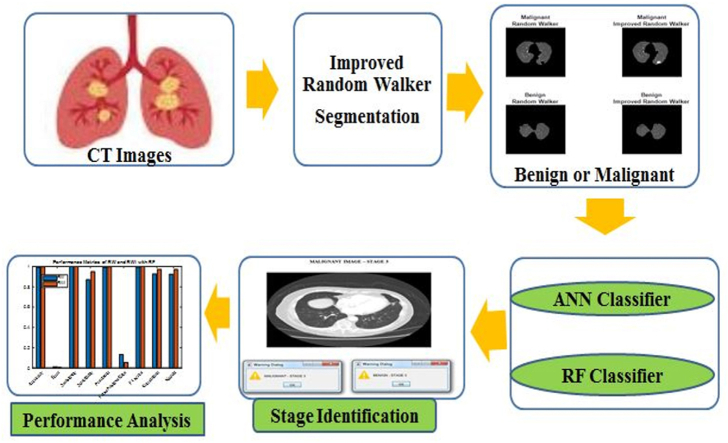

## 1 Introduction

One of the most frequent forms of cancer globally is lung carcinoma, occasionally referred to as simple lung cancer. Because lung cancer can persist for a long time, early detection is crucial for minimizing danger and increasing the chance of survival [1]. Patients' probability of recovery from lung cancer may be greatly increased when pulmonary nodule screening is combined with effective therapy [2]. Computed tomography (CT) is a common imaging technique used to identify and diagnose cancer. Radiologists are the medical professionals who do the clinical examination and categorization of lung nodules as benign or malignant. This is a laborious task with room for error. Furthermore, lung nodules can range in size, contrast, distribution, and shape [3,4]. This makes the effort challenging. The introduction of CAD-based technologies has increased the efficiency and accuracy of medical diagnosis. These tools also assist radiologists to improve the accuracy of their diagnoses through peer review [5,6]. Since there is a wide variety of imaging techniques that can be used on a patient's lungs, automated lung cancer classification forms the challenging tasks that must be completed [7]. In comparison to magnetic resonance imaging (MRI), positron emission tomography (PET), as well as computed tomography (CT) is a less costly non-invasive imaging approach with great spatial resolution [8]. Lung cancer, now thought to be the worst disease in the world, can be diagnosed using a CT scan of the patient.

Therefore, it would make recognize to attempt to develop a system that can categorize and allocate lung nodules based on CT scan images. It goes through a few distinct phases that may be named. Pre-processing may enhance a picture's quality by removing distracting features, which in turn enhances the results of future processes [9]. Image analysis is made easier by using segmentation to isolate foreground objects from their surroundings [10,11]. Extracting properties like intensity, texture, and color from each pixel allows for a one-of-a-kind mathematical representation of the image [[12], [13], [14], [15]]. Then, once objects have been identified, they can be tagged in the image. A support vector machine (SVM) predictor was developed by Alves et al. [16] using CT using and avoiding contrast material. The SVM algorithm placed a premium on texture. To determine whether or not the lumps posed any health risks, support vector machine (SVM) models were developed using data from a variety of sources. After that, the data was sorted using these techniques. To determine whether or not CT scan images indicate lung cancer, Senthil Kumar and colleagues [17] investigated numerous evolutionary image segmentation methods. The adaptive median filter outperformed the mean filter and the median filter during the pre-processing phase. To make the first overwhelming raw image more manageable, four distinct segmentation methods were used. Guaranteed convergence particle swarm optimization (GCPSO), Particle swarm optimization (PSO), clustering using k-means, as well as inertia-weighted particle swarm optimization were among the strategies considered. These systems have an accuracy of 0.885–0.89 up to 0.958 when it comes to classifying data.

It has been shown that a novel method may be used to automatically detect and categorize individual lung cancers by making using CT scan [18]. The CT scan images used as input were cleaned up using a bidirectional filter, thresholding, and morphological segmentation to remove unwanted details. A Bayesian technique was employed to training process execution speed once the size and form of the lung tumors were considered. This allowed us to determine whether or not the tumors included potentially harmful cells. Sharma et al. analyzed DICOM (Digital Imaging & Communications) files including data from CT scans performed to detect lung cancers [19]. As a result, classes numbered 0 and 2 were labelled as “harmless”, classes numbered 4 and 5 as “malignant”, and figures 0.95, 0.88, and 0.84 were given for class 4. Pre-processing procedures may help identify areas of chest CT images that are more likely to contain malignant cells [20]. This may be accomplished by exploring potential hotspots. Features from ResNet and UNet models, both of which are deep residual networks, are inputs to classifiers like random forest and XGBoost [21,22]. Additionally, a method to pinpoint the areas of a CT scan of the lung that are most likely to exhibit signs of cancer was developed [23]. The chance of developing lung cancer was predicted with 84% accuracy by a collection of algorithms, which performed better than individual models. It has been suggested that Deep CNN might speed up the diagnosis process for lung cancer. Their value for the Jaccard similarity coefficient is 0.967, a 0.982 value for the disc similarity coefficient, with a 0.913 value for the area around the operating characteristic curve of the receiver [24]. The use of a Residual Neural Network (RNN) using convolutional learning to secure local characteristics with transformer blocks using self-attention in order to obtain worldwide data was necessary in order to differentiate lung tumors in CT scans [25]. With an AUC of 0.9628 and an accuracy of 0.9292, they were successful. Bruntha et al. offered a mixed categorization strategy for determining the relative safety of lung tumors. This model combines the RNN's hand-crafted features with the histogram's manual adjustments [26]. For determining if a node is malignant or not, Donga provides a modified gradient boost classifier model that can be trained and evaluated using the retrieved attributes [27]. This model may be used to determine whether or not a tumor poses any health risks. The suggested method is checked and double-checked using data from the Lung Imaging Database Consortium (LIDC-IDRI). According to the results of the performance study, the suggested method has a validation accuracy of 95.67 percent, 95.71% accuracy in precision, and 91.71% memory. A total of 0.941 was awarded to the F1.

Despite technological advancements, not everything has been perfected. While most diagnostic tests can only label lung tumors as benign or malignant, doctors may benefit from knowing the subtype. This highlights the critical need for a computer algorithm that can distinguish between various lung cancers. The findings of this research may pave the way for a more precise approach to classifying lung cancers via deploying machine learning methods. Some of these strategies include with the help of artificial neural networks (ANNs) and random forest (RF) classifiers. The proposed research is intended to significantly advance the fields of diagnosis whether lung tumors visible on CT scans are benign or malignant, a modified or enhanced version of the random walker approach is often utilized. The random walker (RW) approach with user-defined seeds is employed during the partitioning stage of the border extraction procedure. The lung tumor is detected in this way. The Local Binary Pattern (LBP) filter may bring back the original colors and patterns in an image. Finally, the accuracy of proposed method is compared with various other classifiers. It has been shown that the RWI-based segmentation and classifier model proposed here outperforms competing approaches.

## 2 Proposed methodology

**Fig. 1:**
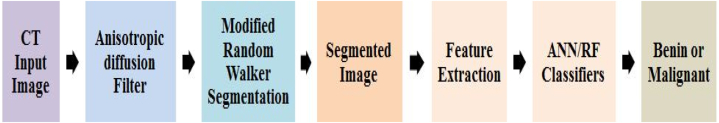
Block illustration of the suggested method for identifying lung cancer.

As part of this work, we present a standalone strategy for segmenting and classifying pulmonary nodules in lung CT scans. Fig. 1 depicts the suggested classification methodology's core organizational features. This model includes four processes: pre-processing, nodule border extraction, feature extraction, and classification. Images of lung cancer from both of these databases can be used as inputs, and following these guidelines to the letter can improve the accuracy and efficiency with which you discover tumors. Using an RWI for persistent segmentation, we may zero in on the precise site of the infection inside the input image. Classifiers are great tools that can aid in making more accurate diagnoses. MATLAB 2018a is currently being modified to incorporate the proposed diagnostic imaging approaches.

RW is used for lung segmentation, followed by feature extraction gets used to getting at information about the lung's texture as well as structure. After that, we use an ANN using a random forest (RF) to categorize the characteristics. The LIDC-IDRI dataset is used throughout the whole process of developing and accessing the proposed model. The LIDC-IDRI dataset contains the largest collection of lung images to date. It includes annotation in XML files and consists of 1018 CT thoracic scans. There are a total of 244,527 images in the LIDC-IDRI collection, which come from 1018 CT scans of 1010 patients. Lung nodules are separated and labelled into five categories in this dataset. Nodules in the lungs that are classed as classes 1 and 2 are regarded to be benign, but nodules in classes 4 and 5 are suspected to be malignant. Lung nodules that have been classified as “class 3” are not considered to be nodules. The DICOM standards are quickly becoming the de facto standard for exchanging and transmitting digital medical records. Since DICOM images typically contain many images at a high resolution, they are typically compressed before being stored or transmitted. Because of this, they tend to be very noisy. This makes the elimination of visual noise an absolute necessity.

### 2.1 Pre-processing

Features of interest in an image are typically analyzed and extracted during the pre-processing phase. To facilitate faster processing, the images of CT scan are converted as jpg. Lung nodule segmentation and classification using this technology have been developed. To do this and improve the quality of the nodules' texture, Artefacts as well as noise within the CT scan images need to be cleaned out. Using an anisotropic diffusion filtering method, these needs can be satisfied.

### 2.2 Anisotropic non-linear diffusion filter

An adaptive method for decreasing noise keep finer details and crisper edges is provided by the anisotropic nonlinear diffusion filter. Therefore, the pulmonary nodule borders can be more accurately defined using the random walker method. This filter improves the image's texture quality as well, which facilitates precise texture data extraction. Preprocessing the image yields the same characteristics. The goal of anisotropic non-linear diffusion (also called Perona-Malik diffusion) is to reduce the amount of noise in an image without compromising any of the image's essential characteristics, such as sharp edges and clean lines. Using a constant diffusion coefficient, noise in digital images can be eliminated with no perceptible blurring to the edges.

**Table:**
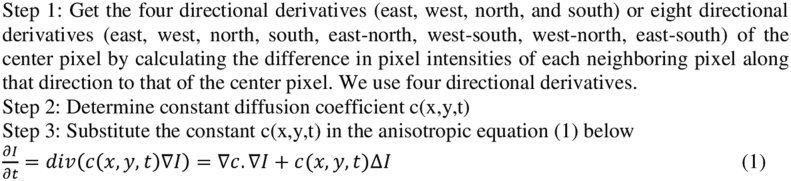
Algorithm Anisotropic diffusion

where Δ is their Laplacian, ∇ is gradient, div(…) indicates the divergence operator as well as c(x,y,t) represents diffusion coefficient.

### 2.3 Random walker segmentation and improved random walker segmentation

When it comes to the challenge of picture segmentation, the semi-supervised RW methodology excels. Instead of representing the image as a matrix, this technique converts it into a graph, with the pixel intensities assigned to individual nodes (called seeds). An edge extends from each node to the next-door neighboring node. One seed is used to focus on the nodule (the subject) and the second seed is used to fill in the rest of the lung (the background) in the final image. The probability that an RW pixel will reach each seed first determines whether or not it is associated with the background or foreground label. This possibility is represented by probability. By solving a similar electrical circuit with “0” and “1” as seeds for the voltages at each node, we may determine the answer to this RW issue.

During random segmentation, the only thing that changes how the weights are created is the images' brightness. In contrast, RWI uses both the image intensities and the retrieved LBP texture features to generate weights. The current pixel's value is used as a threshold in LBP, making it a powerful texture descriptor. Images' textures can thus be described using LBP. LBP descriptors are very effective in characterizing both the local spatial patterns and the grayscale contrast of an image. The nodule produced by the segmentation is then utilized for further investigation once the picture has been broken down. The following are some of the main advantages of the RWI method: It's simple to implement, (a) can split the picture into several regions depending on the number of seed labels given, and (b) can deal with intricate boundary configurations. In this concept, each pixel represents a node in a network.

**Table:**
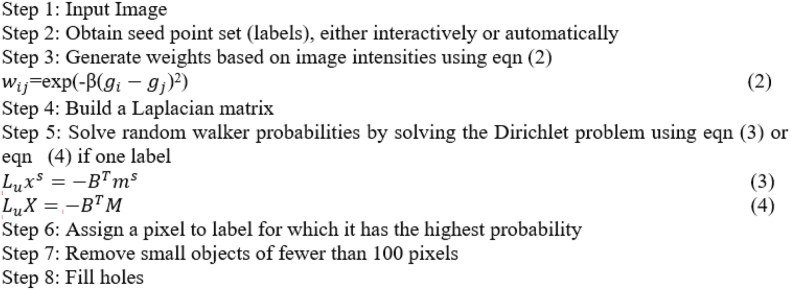
Algorithm: Random Walker

**Table:**
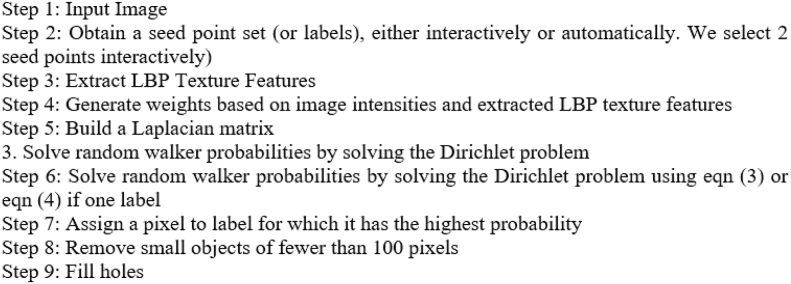
Algorithm: Improved Random Walker

The LBP filter is a popular method for encoding and categorizing pictures in machine-learning models. It is a basic graphical operator that assigns a binary value to each pixel in an image by thresholding the region around it. The texture operator is surprisingly potent for how simple it is. It has progressed into a useful approach for a variety of uses thanks to its unequal strength and effective use of computation. As a result, it has replaced structural and divergent statistical texture analysis as the preferred method for fusing disparate data sets. As a consequence, the technique enhances performance in a variety of applications, including facial expression analysis, object recognition, and texture classification. Here the filter extraction is extended by a 3 × 3 sliding window, extending the LBP code. By comparing the thresholds of the window's outermost and innermost pixels, this LBP code is created.

### 2.4 Feature extraction

The most common basis for extracting image features used as predictors in classification and regression are intensity, shape, and texture. These qualities are taken out of visual data. To do RW segmentation features like intensity and texture must be extracted. Additionally, the Reisz wavelet coefficients are computed to extract the texture data. The classification of tumor types linked to cancer is then aided by the use of this data.

### 2.5 Classification

To complete a diagnosis, it is necessary to extract and categorize features. The goal of every classifier is to discover connections between features in the input data that may be used to infer the class to which the data belongs. All classifiers should be able to do this. The three most significant parameters used to evaluate a classifier are its accuracy, specificity, and sensitivity [15]. For this article, “sensitivity” refers to the percentage of false-positive malignant classifications while “accuracy” refers to the percentage of correctly recognized images. The specificity measures the proportion of correctly tagged images that are safe to see, whereas the area under the curve is a metric that allows for the ideal models to be selected. Both of these ideas can be expressed as a percentage of safe pictures. Values closer to 1 indicate more precise categorizations, however, it can take on any number between 0 and 1. The enhanced random walker method segments the images, and then those segments are put into an ANN classifier and an RF classifier. The most robust, accurate, and flexible algorithms are the RF and ANN ones, and they may be used for both regression and classification. Predictions from the RF classifier are a composite of the findings from numerous regression trees. Each connection between nodes in an artificial neural network (ANN) is given a value or weight. Implementing the findings of these studies has the potential to significantly cut down on the total cost of staging by minimizing the amount of labor involved and the number of mistakes made by humans. This will trigger a dramatic shift in the medical industry by increasing the effectiveness of staging. When being trained, RF uses many different decision trees before picking the one with the smallest error rate. Because of this, it can improve its accuracy. The Alignment, Enclosure, and Solidity features are part of the shape features; the Contrast features are part of the GLCM features; the Homogenization features are part of the GLCM features; the Cluster Prominence features are part of the GLCM features; the Cluster Shade features are part of the GLCM features; and the Dissimilarity features are part of the GLCM features. By first removing outliers in intensity, the Single-Level Discrete 2-D Wavelet Transform can yield normalized values for the Principal Component Coefficients.

## 3 Results and discussion

**Fig. 2:**
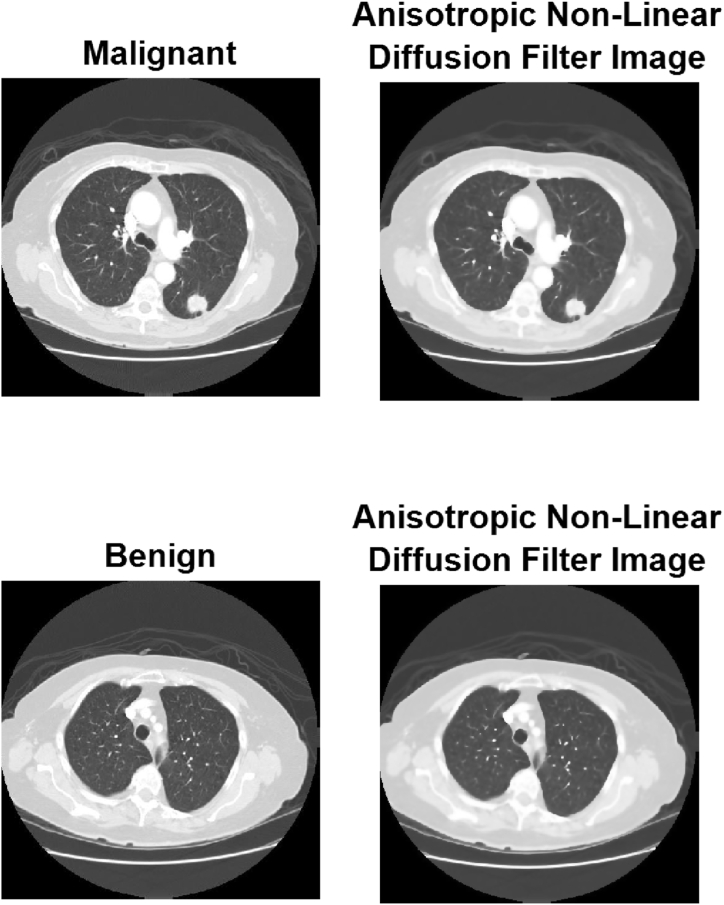
Anisotropic non-linear filtered image after preprocessing.

DICOM images are frequently compressed during storage and transmission due to the large number of high-resolution images they include. The first step in processing any DICOM image from the LIDC archive is to convert it to a Jpeg file format. Once this is complete, a training dataset containing 534 images and a testing dataset containing 150 images of benign and tumorous lung cancer will be obtained. Select a sample image from the repository and have it grayscaled by having its hue, saturation, and brightness stripped away. Representativeness reduction is a method employed by autonomous image processing systems to both improve the quality of the images they create and reduce their reliance on human input. There are two primary categories of contrast amplification methods: frequency-domain methods and spatial-domain methods. The presence of this element will cause the amplitude of lower frequencies to drop. An anisotropic diffusion filter is applied as preliminary processing to the image to eliminate unwanted noise. After the lung image has been segmented using a refined random walk method, the region of interest is extracted. Feature extractions with the help of the textural feature extraction technique of pulmonary nodules, which takes into account the continuity of the image slices. In the final step, the classifier algorithm is used to identify and categorize the pulmonary nodules. Fig. 2 displays the processed, anisotropic, non-linear filtered image.

**Fig. 3:**
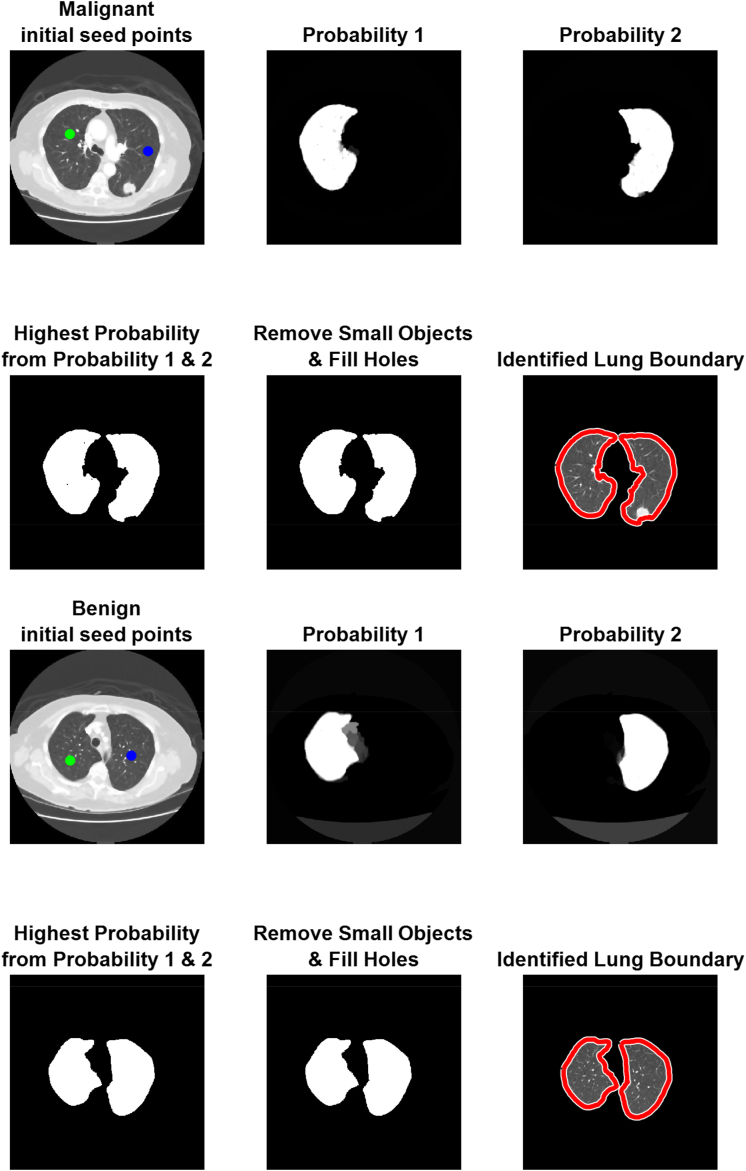
The process of RWI for malignant and benign images.

Lung nodule margins are segmented and extracted so that RWI can be used to see if it improves segmentation performance. The segmentation block is fed CT images of lung nodules that have already had their boundaries removed. Fig. 3 displays the original CT scan with the segmented result.

**Fig. 4:**
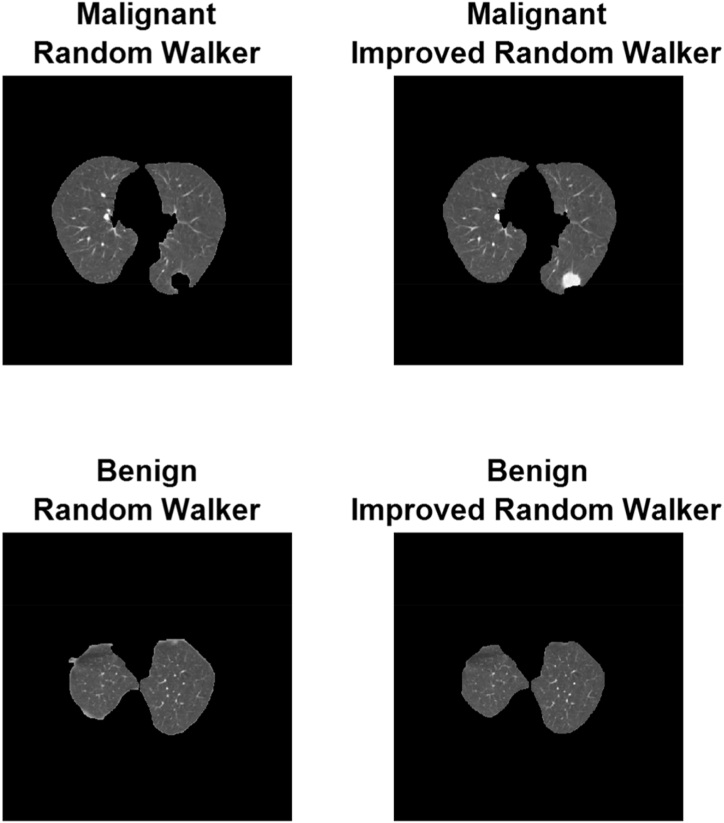
Outputs of RW and RWI for malignant and benign images.

Most frequently, feature extraction is used for contour identification and image distribution. Both of these programs will come in handy in many situations. This quality appears in several different manifestations throughout the picture. The binarization technique has multiple applications, including cancer detection and image enhancement by highlighting focal points. It appears that extracting features was crucial in recognizing and categorizing a wide variety of specified geometries. The degree of binarization can be determined by counting the number of monochrome or grayscale pixels present in the image. The demonstration that normal tissue images contain a much higher proportion of black pixels than aberrant lung images containing white pixels is important to the binarization design and architecture. If the percentage of dark-to-light areas in an image is significantly out of the norm, we characterize it as abnormal. The quality of the aforementioned binary images was further diminished by subjecting them to morphological processing, which is typical of binary images. The second process required expanding the image to fill in the spaces where pixels normally wouldn't be visible from outside the image's context. Finally, a segmentation marker in the shape of a disc is applied to the image pixels to identify the segmented region. Results of RW and RWI on benign and malignant pictures are shown in Fig. 4.

The discrete 2-D wavelets' main components are used as the single level of derivation for the 13 form characteristics. Among the 13 recovered form qualities shown in Table 1 are Area, Convex Area, Orientation, Centroid, Equiv Diameter, Eccentricity, Perimeter, Extent, Extrema, Euler Number, Major and Minor Axis Length, and Solidity.

**Table 1:** Shape-based Parameters for the segmented region.

Sl no	Constraints	Malignant	Benign
1	Area	21,895	18756.5
2	Solidity	0.8371	0.9174
3	Centroid	254.799	256.551
4	Perimeter	674.92	543.145
5	Convex Area	26700.5	19,493
6	Orientation	−1.0373	88.1793
7	Eccentricity	0.8485	0.7464
8	Minor Axis Length	131.3741	123.7102
9	Equiv Diameter	166.2869	151.2964
10	Major Axis Length	246.2452	194.1435
11	EulerNumber	1.0	1.0
12	Extrema	241.9735	255.2583
13	Extent	0.6458	0.7106

The 7 GLCM features extracted are Cluster Shade, Entropy Energy, Contrast, Cluster Prominence, Homogeneity, as well as Dissimilarity, depicted as Table 2.

**Table 2:** GLCM-based texture features.

Sl no	GLCM parameters	Malignant	Benign
1	Contrast	0.0344	0.0538
2	Dissimilarity	0.0527	0.0319
3	Entropy	0.6658	0.5239
4	Cluster Shade	15.5645	8.6842
5	Energy	0.7271	0.7757
6	Cluster Prominence	131.6972	48.2474
7	Homogeneity	0.9984	0.9895

The various intensity variables and their corresponding values are listed in Table 3. Using the Single-level Discrete 2-D Wavelet Transforms principal Component Coefficients, intensity parameters depicted in Table 2 are used. Three other ways to assess dispersion are smoothness, kurtosis, and skewness. The visual coherence of the image affects IDM. The weighting algorithm makes sure that non-uniform regions only contribute a small amount to the IDM. The IDM value of non-homogeneous images thus decreases, whilst the value of homogeneous images increases.

**Table 3:** Intensity features.

Sl no	Intensity features	Malignant	Benign
1	Mean	0.0108	0.0107
2	IDM	3.4347	3.4673
3	Standard Deviation	0.0957	0.0957
4	Smoothness	0.9673	0.9659
5	Skewness	3.699	3.7844
6	RMS	0.0958	0.0958
7	Kurtosis	65.3637	62.7947
8	Variance	0.0126	0.0136

**Fig. 5:**
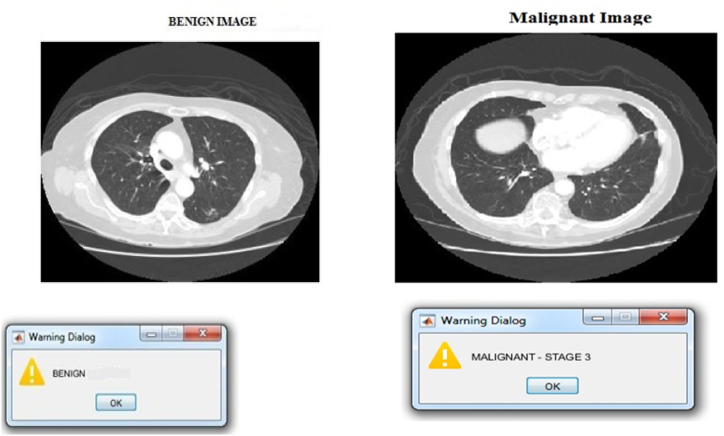
Stages of benign and malignant images.

The aforementioned estimated parameters served as the basis for the derivation of the 28 features, which included 7 8-intensity features, GLCM texture features, as well as 13 shape features. The aforementioned procedure is applied to each of the 534 images contained in the training database to generate the features that are subsequently utilized in the training of the ANN and RF-based classifier. The developmental phases of benign and malignant images are depicted for your viewing pleasure in Fig. 5.

Nine performance metrics, including Specificity, Accuracy, Sensitivity, False Positive Rate, Precision, F1 Score, Mathews Correlation Coefficient, as well as Kappa-Cohen's Kappa, are used to assess the classification efficacy of the predicted methodology parameters of RW as well as RWI using ANN and RF. The Two-Class of Confusion Matrix allows for the possibility of a True Positive (TP), False Positive (FP), False Negative (FN), and True Negative (TN). Eq. (5) displays the resultant equation.

(5) Acc = (TP + TN)/ (TP + FP + TN + FN)

Sensitivity (Se) is the fraction of node variables correctly predicted, shown in Eq. (6), and Specificity (Sp) is calculated as the images correctly predicted shown in Eq. (7).

(6) Se = TP/ (TP + FN)

(7) Sp = TN/ (TN + FP)

The false positive ratio (FPR) is the percentage of pixels incorrectly labelled as nodes, shown in Eq. (8) and the false negative ratio (FNR) appears to be the percentage of pixels with incorrect values, shown in Eq. (9) [15].

(8) FPR]FP/ (TP + TN)

(9) FNR]FN / (TP + TN)

The overlapping value is an indicator of similarity that is a reproduction of how the principles' subdivision result binds the truth, shown in Eq. (10).

(10) Overlap = TP/ (TP + FP + FN)

where True Positive = exactly found number as nodule pixels.

False Positive = incorrect found number as nodule pixels.

False Negative = number of incorrect identifications as contextual images.

True Negative = the number of exact identifications as background pixels.

Five calculation measures are given a score between 0 and 1. The better the performance of a subdivision, the lower the FPR and FNR.

**Fig. 6:**
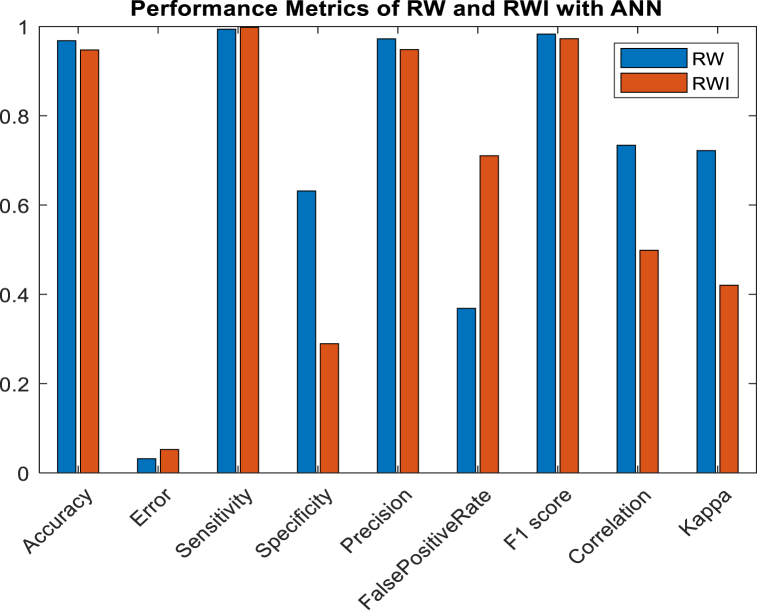
Performance metrics analysis of RW and RWI with ANN.

**Fig. 7:**
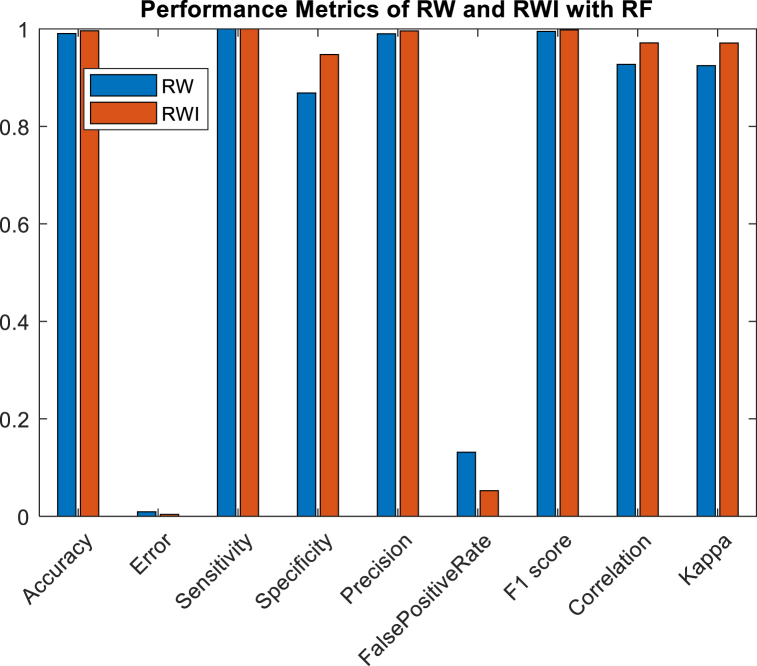
Performance metrics analysis of RW and RWI with RF.

Multiple classes are labelled and predicted to generate a confusion matrix [5]. Accuracy, error, Sensitivity (Recall or TP rate), Specificity, Precision, FPR-FP rate, F1 score, MCC-Matthews correlation coefficient, and kappa-kappa Cohen's are calculated to evaluate the accuracy of RW and RWI using ANN are illustrated in Fig. 6, and performance evaluation of RW and RWI with RF is depicted as Fig. 7. The performance evaluation values of ANN and RF are depicted in Table 4.

**Table 4:** Performance metrics analysis values of RW and RWI with ANN.

Sl no	Performance metrics analysis	RW	RWI	RW	RWI
		ANN		RF	
1	Accuracy	96.8255	94.7656	99.0367	99.6535
2	F1_score	98.3141	97.2945	99.4895	99.7898
3	Error	3.1385	5.2344	0.9336	0.3475
4	False positive rate	36.8341	71.0256	13.1759	5.2362
5	Sensitivity	99.3592	99.7894	99.9889	99.9889
6	Mathews Correlation Coefficient	73.3889	49.8362	92.7339	97.1462
7	Kappa	72.2208	42.0285	92.4859	97.0852
8	Precision	97.2857	94.8366	99.0030	99.5894
9	Specificity	63.1759	28.9744	86.8241	94.7458

The rows of any multiplication chart stand in for the target domain in a confusion matrix, while the columns stand in for the output class and the target class names stand in for the target domain. The cells on that diagonal display information relating to precision. The labels denoting the discoveries, which were supposed to be placed above the off-diagonal cells, were positioned incorrectly. The frequencies of the right and wrong answers can be compared using uncertainty matrices. On the diagonal of the relevant matrices, green squares denote correct classifications, while red squares denote incorrect ones. The percentage of total observations and the number of interpretations are shown separately in each cell. On the far right of both maps are columns indicating what fraction of expected occurrences was tagged properly and what fraction were mislabeled. Accuracy, often known as the positive predictive score or the fraction of false positives, is a popular criterion. The right and incorrect case classification rates are displayed at the bottom of the matrix. All expected categories are taken into account here. There are two ways to quantify memory recall. The true positive and false negative rates are as follows. The accuracy of the measurement is depicted by the percentage of white cells in the bottom right corner of the matrix.

**Fig. 8:**
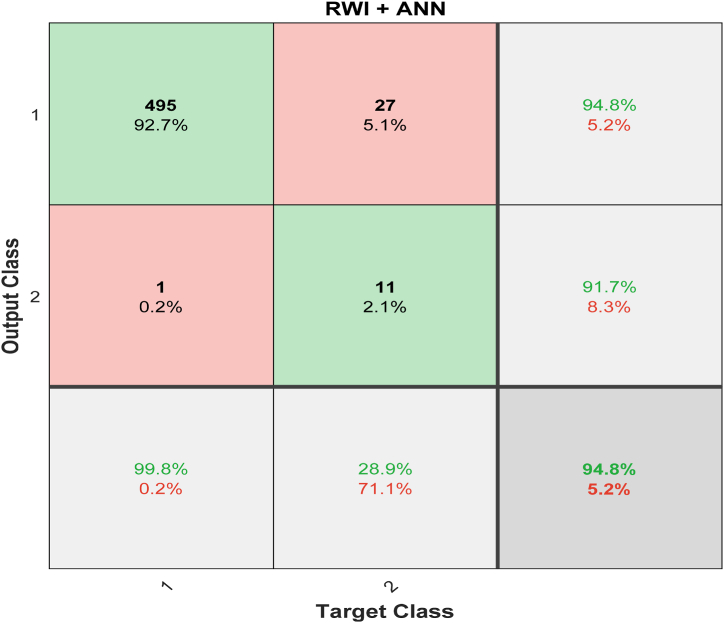
Confusion Matrices for RWI with ANN classifier.

The confusion matrix and the expected and observed classifications are shown in Fig. 8. The total number of duplicated arrangements and the percentage of duplicated arrangements produced by the trained system are both displayed in the top two diagonal cells, respectively. Out of 534 images, only 495 could be verified as safe (the true positive). After comparing all 534 images side by side, we found that 94% of them are comparable. The 27 benign cases that have been successfully identified so far (true negatives) can be deemed to be the same. It is only possible to distinguish this image from any other in 5.1% of cases. Whenever even one of the potentially cancerous images is incorrectly classified as benign, false-positive rates increase to 0.2%. Similarly, 11 images (2.1% of the total) that are not malignant are mistakenly classified as such. This mistake is referred to as a “false negative”. With a negligible 5.2% margin of error, RWI's ANN classifier, which it utilizes for classification, achieves an outstanding 94.8% accuracy.

**Fig. 9:**
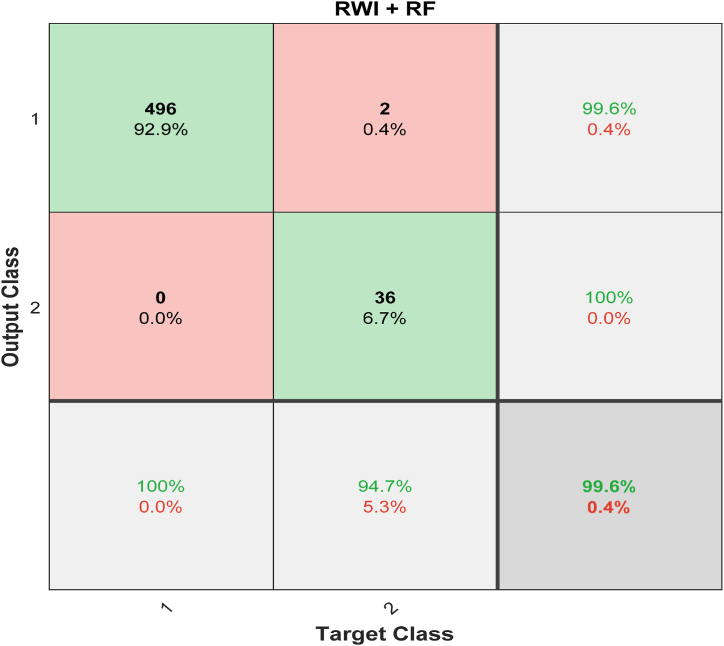
Confusion Matrix for RWI with RF classifier.

**Fig. 10:**
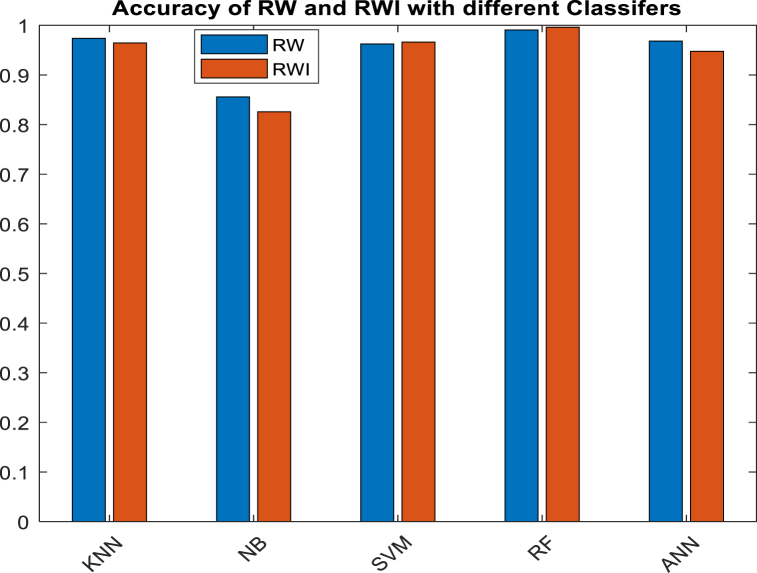
**A**ccuracy comparison of RW and RWI with various classifiers.

Fig. 9 shows the input class, the predicted output class, and the confusion matrix of RWI with the RF classifier. Only 496 of the 534 images (91.9%), or the “true positives,” can be safely described as harmless. There were also two cases (true negatives) that did not meet the standards for cancer. Only 0.4% of other images are similar to it. None of the pictures that show cancer were mistakenly labelled as not showing cancer. On the other hand, 6.7% of all the data has to do with false positives, which is when non-cancerous images are wrongly labelled as cancerous. When an RF classifier is used, RWI can get 99.6% accuracy with a 0.4% mistake rate. Fig. 10 shows the results of a comparison between how accurate RW and RWI are and how accurate KNN, NB, SVM, RF, and ANN are as classifiers. This shows that the proposed model is better.

## 4 Discussion

Accurate lung image segmentation is especially crucial in detecting lung lesions and quantitatively analyzing local lesion information. Our investigation revealed the suggested method's robustness in the face of varied CT reconstruction configurations and the presence of lung pathologies. Problems with lung image segmentation are mostly from irregularities around the fissures, which cause failures in fissure recognition or substantially altered lobar forms that cannot be reported. Our method is an automated segmentation model that combines the benefits of fully-automatic segmentation and reduces the impact of the aforementioned problems on lung lobe segmentation accuracy. This will enable for the evaluation and comparison of the performance of different phases of the algorithm, allowing for the hybrid method. Image pre-processing, segmentation, feature extraction, image classification, and performance are the five stages in which the methodologies have been classified and implemented. The simulation results are achieved by varying four parameters: accuracy, F1 score, precision and recall. The obtained results show that multilayer perceptron or neural networks can be used for detection and classification of lung cancer CT scan images with excellent accuracy and precision when compared to other methods [16,18,25].

The two classifiers were all trained using the previously discussed GLCM feature set, which includes all 28 features. A subset of the complete dataset was then used to construct performance evaluation using the nine parameters indicated above for measuring the performance of a classifier. This was done to ensure a consistent result without the risk of overfitting the dataset [19,20]. The effect of each feature on the overall accuracy percentage, F1 score, precision, and recall for each classifier was measured by removing that feature from the feature set and then training the classifier on the remaining features in the feature set out of 7 GLCM features extracted in the feature extraction stage. The effect of this study is pretty clear for segmentation of the lungs, which is the precision of the expert's time. In the future, this research could continue to remove the suspicious area of the lungs with professional experience to boost interaction efficiency. This innovation may have a major impact on the worldwide rate of lung cancer rate due to its ability to detect lung tumors in their earliest stages when they are most amenable to being avoided and treated. This method is useful because it provides more information and facilitates quick, precise decision-making for doctors diagnosing lung cancer in their patients.

RF enhanced detection in low dose CT by integrating a model-based local shape analysis with data-driven local contextual feature learning [9]. The technique was trained to acquire and merge a subset of these basic elements into distinctive orientation invariant contextual characteristics, and subsequently classify nodule candidates. Through the implementation of this approach, the algorithm achieved a sensitivity rate of 80% in Ref. [9]. ANN approach that was trained using the LIDC-IDRI database utilized 3D geometric and statistical attributes to form a voting mechanism. During the implementation of this method, the algorithm achieved a sensitivity of 89.4% [26]. Reference [27] utilized a straightforward rule classifier and attained a cumulative accuracy of 70.53%. ANN classifier is used to enhance accuracy and minimize the number of FP [17]. Reference [11] had the greatest sensitivity, specificity, and AUC accomplished via a RF classifier. The results of proposed methodology are satisfactory, based on the classifier used in the initial stage of the classification process. These contributions should progress beyond the LIDC-IDRI database and be duly considered for the implementation of this technology in clinical practice.

The limitations of this research may be restricted by variations between datasets and the diversity of tumors inside an individual. Multiple studies need to be conducted to address the disparity between the conventional method and personalized evaluation, resulting from an inadequate methodology. Obtaining a suitably large and diverse dataset for lung cancer diagnosis can be problematic due to privacy concerns and the rarity of specific types of lung cancer. The efficacy of these algorithms may vary depending on the specific characteristics employed, the data's quality, and the general architecture of the detection system. Employing various algorithms and ensemble methods can be a strategy to improve overall performance. Hence, future research in this domain will focus on devising a technique to incorporate supplementary variables in order to improve the performance of the model.

## 5 Conclusion

In this research, we use the RWI method to construct a robust and time-saving framework model for lung nodule classification in CT scans. The CT scan pictures are greatly enhanced during the pre-processing step. The Riesz wavelets provide a solid basis for image representation for feature extraction, and the RWI algorithm can successfully segment the CT scan picture to the required depth. The suggested classifier can accurately determine whether lung nodules are benign or cancerous. Potentially significantly affecting the global lung cancer burden is this innovation's potential to detect lung tumors in their early stages, when they are most receptive to prevention and therapy. This is done to detect lung cancer as early as possible. This method can help doctors diagnose lung cancer earlier and more accurately, and it can also improve patient care by facilitating faster and more informed decision-making. The proposed technique has an accuracy of 94.8% for diagnosing lung cancer, while the RF classifier has an accuracy of 99.6%. This implies that the RF classifier is highly accurate for lung tumor classification using RWI.

## Ethics declaration

Review and approval by ethics committee was not needed for this study because no animal study is included in this research and this research utilized publicly available image dataset.

## Data availability

Data will be available on request to corresponding author. Raw data is not available in public repository as it is authors Ph.D. research topic.

## CRediT authorship contribution statement

**Sneha S. Nair:** Writing – original draft, Visualization, Validation, Software, Project administration, Methodology, Investigation, Formal analysis, Data curation, Conceptualization. **V.N. Meena Devi:** Supervision, Data curation. **Saju Bhasi:** Writing – review & editing, Supervision.

## Declaration of competing interest

The authors declare that they have no known competing financial interests or personal relationships that could have appeared to influence the work reported in this paper.
